# Dosing Challenges and Benefits of Vericiguat in a Patient With Decompensated Heart Failure: A Case Report

**DOI:** 10.7759/cureus.48065

**Published:** 2023-10-31

**Authors:** Mario Speranza-Sánchez, Esteban Zavaleta, Sonia Sancho-Zumbado, Juan Carlos Elizondo-Urrutia, Alonso Quirós-Romero, José Chaverri-Fernández, Jonathan García-Montero, Sebastián Arguedas-Chacón

**Affiliations:** 1 Heart Failure Program, Hospital Clínica Bíblica, San José, CRI; 2 Pharmacy, Hospital Clinica Biblica, San Jose, CRI; 3 Faculty of Pharmacy, University of Costa Rica, San José, CRI; 4 Pharmacology, Toxicology and Drug Dependence, Universidad de Costa Rica, San Jose, CRI; 5 Pharmacy, Hospital Clínica Bíblica, San José, CRI; 6 Research, Hospital Clínica Bíblica, San Jose, CRI

**Keywords:** hypotension, cyclic gmp, soluble guanylyl cyclase, heart failure, vericiguat

## Abstract

Vericiguat has emerged as a promising add-on therapy for decompensated heart failure with reduced ejection fraction (HFrEF) patients requiring hospitalization or IV diuretic administration. In the VICTORIA trial (Vericiguat Global Study in Subjects with Heart Failure with Reduced Ejection Fraction), vericiguat was demonstrated to significantly reduce mortality and hospitalization rates. However, the effect of vericiguat on patients receiving SGLT2 inhibitors remains uncertain. In this report, we present a complicated case of dilated heart failure receiving low doses of foundational therapy due to a patient’s intolerance but still experiencing recurrent hospital readmissions. Following six months of low-dose vericiguat as an add-on therapy, the patient exhibited important improvements in various clinical parameters, including cardiac and renal function. Nonetheless, further investigation is crucial to substantiate the additional benefits of combination therapy. These findings provide further evidence for the potential benefits of vericiguat when treating HFrEF.

## Introduction

Heart failure with reduced ejection fraction (HFrEF) is a condition with a growing prevalence among older adults [1,2]. While therapeutic advances have managed to slow the progression of the disease and improve the quality of life of patients, hospitalization rates remain high, reaching 32-44% [3]. In Latin America, the prevalence of HFrEF is 1%, and hospital readmission rates are approximately 31% to 35% [4]. These hospitalization rates are a significant predictor of morbidity and mortality and have a direct impact on the deterioration of patients' quality of life as well as hospitalization costs. Therefore, it is crucial to continue researching therapies that can reduce these rates. One promising new treatment for HFrEF is vericiguat [1-4].

Vericiguat is a novel guanylate cyclase stimulant indicated in HFrEF. The VICTORIA (Vericiguat Global Study in Subjects with Heart Failure with Reduced Ejection Fraction) trial studied this medication in patients with recurring hospitalization or intravenous diuretics with a creatinine clearance of 15-30 mL/min/1.73 m^2^ [5]. By stimulating the guanylate cyclase, vericiguat promotes the cyclic guanosine monophosphate (cGMP) pathway, which increases nitric oxide signaling. This results in a reduction of left ventricle remodeling, improvement of vascular and diastolic function, as well as a decrease in fibrosis and inflammation. Within 12 weeks, there’s a slight improvement in pro-BNP levels and left ventricular ejection fraction (LVEF) and a significant improvement in the quality of life [5-7].

The effects of vericiguat when added to foundational therapy, non-specific beta blockers, SGLT2 inhibitors, angiotensin and neprilysin inhibitors (ARNI), and mineralocorticoid receptor antagonists remain unclear because the VICTORIA trial included very few patients receiving SGLT2 inhibitors, so results in this matter are not significant [5]. This case report presents the first instance of a patient receiving vericiguat for six months in Costa Rica. The patient receives low doses of the foundational therapy due to intolerance. The introduction of vericiguat in this patient required dose adjustment in chronic therapy due to hypotension during its titration, and the impact of vericiguat on the overall treatment in patients with pre-existing therapy remains an area of interest for further investigation.

## Case presentation

Before referral

A 72-year-old male patient with ischemic cardiomyopathy presented with dilated heart failure, a reduced LVEF of 24%, and elevated pro-BNP levels of 17,406 pg/mL. The patient had NYHA class III symptoms: grade III cardiomegaly, atrial fibrillation, dyslipidemia, stage IIIb chronic renal failure due to cardiac causes, Child-Pugh B liver disease, and hypothyroidism. The patient was being treated with sacubitril/valsartan 24.3/25.7 mg twice daily, eplerenone 25 mg once daily, bisoprolol 2.5 mg once daily, dapagliflozin 10 mg once daily, and furosemide 80 mg three times a day. Despite this regimen, the patient continued to experience severe congestion and required frequent hospitalizations for drainage.

On referral

Over the course of two months, the patient was hospitalized twice due to decompensated heart failure. The Heart Failure Program (HFP) approached the patient to enroll him in the program and provide ongoing care. Due to his hospitalizations for cardiovascular causes, the patient was a candidate for vericiguat treatment. The HFP cardiology physician contacted the Health Ministry of Costa Rica to request endorsement for using vericiguat in this patient, even though the drug use is not yet approved in the country. Upon admission to the HFP, additional tests were conducted, which revealed a six-minute walk distance of 480 m, a Kansas Score of 50 points, and a Barthel Score of 90 points.

**Figure 1:**
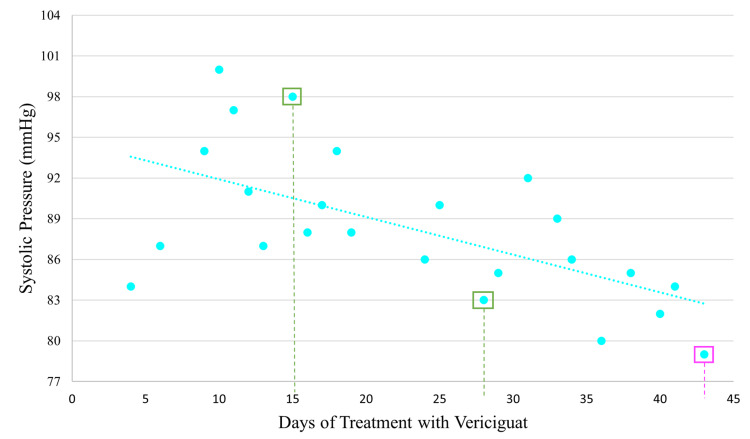
Evolution of systolic blood pressure in the period of vericiguat titration. Green boxes contain dots that indicate when the recommended dose escalation by the manufacturer was carried out. The magenta box represents the time when the patient experienced a hypotensive event, leading to the decision to decrease the dose.

Upon initiating vericiguat treatment, the manufacturer’s dose recommendations were followed, starting the patient on a low dose of 2.5 mg, then doubling every 15 days until reaching a target dose of 10 mg once daily. Given the potential risk of hypotension, systolic blood pressure was closely monitored and used as a parameter for dose adjustments, with a target range of 90-100 mmHg. This patient has a low blood pressure baseline, so the tendency to hypotension when using heart failure medication is recurrent when initiating vericiguat, and doubling its dose caused hypotension within three days of each dose titration, but the patient tolerated the medication well throughout the titration period, despite the progressive decrease in systolic pressure, which is illustrated in Figure 1.

The target dose of 10 mg of vericiguat once daily was achieved after 28 days of treatment. The titration days are marked with a green square and pointed line in Figure 1. Despite this achievement, the patient experienced a hypotension event with a systolic blood pressure of 79 mmHg on day 43, for which the vericiguat dose was reduced to 5 mg once daily and has been maintained since then. This event is marked with a magenta square and a pointed line. To avoid hypotension, the ARNI dose was also decreased, and it is currently administered as 24.3/25.7 mg in the morning and 12.15/12.85 mg at night. Additionally, the patient developed bradycardia, and bisoprolol had to be suspended until the patient was more stable with vericiguat. One month later, bisoprolol was reintroduced at a low dose of 1.25 mg once daily. Dose changes for heart failure medication are illustrated in Table 1.

**Table 1:** Daily dose of cardiovascular medication upon HFP admission and current daily dose of cardiovascular medication with six months of vericiguat add-on therapy. ^a^Reduced dose to avoid hypotension or bradycardia. ^b^Reduced dose because of decreased requirement. HFP: Heart Failure Program.

Drug	Daily dose upon admission (mg)	Current daily dose (mg)
Vericiguat	0	5
Sacubitril/Valsartan	48.6/51.4	36.45/38.5^a^
Bisoprolol	2.5	1.25^a^
Furosemide	240	40^b^
Dapagliflozin	10	10

The administration of vericiguat in this patient with dilated heart failure and reduced ejection fraction significantly improved several clinical parameters. Notably, the patient experienced a significant decrease in fluid congestion and a reduction in diuretic requirements, leading to an improvement in quality of life. The patient has been asymptomatic for over three months and has not required hospitalization since initiating vericiguat add-on therapy. In addition, improvements were observed in the patient’s NYHA class, pro-BNP levels, renal function, and quality of life scores. These results are illustrated in Table 2.

**Table 2:** Renal profile, pro-BNP, LVEF, and NYHA class evolution upon admission to the HFP and current values with six months of vericiguat. Cr: creatinine; BUN: blood urea nitrogen; LVEF: left ventricular ejection fraction; pro-BNP: N-terminal pro-b-type natriuretic peptide; NYHA: New York Heart Association. ^a^Following a 6-month course of vericiguat treatment.

Parameter	Base value	Mid-term value	Current value^a^
Cr	2.42 mg/dL	2.31 mg/dL	1.65 mg/dL
Creatinine clearance	32 mL/min/1.73 m^2^	29 mL/min/1.73 m^2^	44 mL/min/1.73 m^2^
BUN	43 mg/dL	43 mg/dL	25 mg/dL
LVEF	24%	34%	30%
Pro-BNP	17,406 pg/mL	13,537 pg/mL	10,239 pg/mL
NYHA Class	III	II	I
Barthel Score	90 pts	95 pts	100 pts
Kansas Score	50 pts	54 pts	60 pts
Six-minute-walk	480 m	500 m	570 m

## Discussion

In the pathology of decompensated heart failure, there is a notable inhibition of the guanylate cyclase and nitric oxide pathways. Therefore, the mechanism of action of vericiguat, which targets and enhances these pathways, can play a crucial role in controlling the disease. By promoting cGMP production, vericiguat facilitates the dilation of blood vessels, reduces cardiac workload, and improves cardiac function [5,6]. As a result, the use of vericiguat has been shown to be effective in managing decompensated heart failure, particularly in patients with reduced ejection fraction [8].

In the VICTORIA trial evaluating the efficacy of vericiguat, a positive correlation between dose and reduction in pro-BNP levels was observed. Despite our patient receiving a dose of 5 mg once daily, he has a pro-BNP level reduction of 7.167 pg/mL. Additionally, an increase in LVEF was found in this trial and observed in our patient; these two parameters represent an improvement in our patient’s condition. However, it should be noted that these improvements, although promising, were not statistically significant regarding disease progression. Nonetheless, using vericiguat has demonstrated significant benefits, such as reducing mortality from cardiovascular causes and decreasing hospitalization rates among patients with HFrEF [5]. Furthermore, the drug has also been associated with improving the overall quality of life, which was also observed in our patient [6,7]. Subsequent studies to the VICTORIA trial have demonstrated that patients with an NT-proBNP level below 8000 pg/ml experience a reduction in cardiovascular mortality or initial hospitalization for heart failure, suggesting that the treatment is most beneficial for patients with less advanced HFrEF [9,10].

The 2021 edition of the heart failure guidelines was the first to mention vericiguat, albeit only briefly as an emerging treatment option, despite the positive results of the VICTORIA trial. The 2022 heart failure guidelines recommend the addition of vericiguat to guideline-directed medical therapy for patients with chronic heart failure and a reduced ejection fraction (EF < 45%) who remain symptomatic or are at high risk of hospitalization despite optimal treatment with other medications [11,12].

Vericiguat has been shown to have a favorable safety profile for renal function, with no reported negative effects in clinical trials. However, the impact on renal function improvement was not investigated in the VICTORIA trial, although it only included patients with creatinine clearances of 15-30 mL/min/1.73 m^2^ [5]. A study evaluating the correlation between vericiguat efficacy and baseline and subsequent renal function changes concluded that vericiguat does not affect the progression of kidney disease in patients with severe heart failure. Additionally, vericiguat may benefit patients with severe heart failure, regardless of their baseline kidney function [13].

Our patient, who had preexisting stage IIIb chronic renal failure, demonstrated a significant improvement with a decrease in severity approaching stage IIIa classification. This suggests that future studies may benefit from investigating the impact of vericiguat on renal function in patients with more advanced renal failure.

It is worth noting that our patient is receiving a lower dose of vericiguat, 5 mg once daily, compared to the 10 mg once daily studied in the VICTORIA trial (median dose 9.2 mg) [5], and our patient is also concurrently receiving an SGLT2 inhibitor, which may influence the patient's clinical course differently than in the trial. Therefore, further investigation into the use of low-dose vericiguat as an add-on therapy to the foundational therapy is necessary.

## Conclusions

Vericiguat has demonstrated its potential for managing decompensated HFrEF as an add-on therapy. This case report shows that vericiguat, in conjunction with fundamental therapy, could provide other clinical benefits in terms of renal and cardiac function to patients with dilated heart failure when administered in low doses for a period of six months. Furthermore, additional research is needed to determine and analyze the potential additional benefits of combination therapy in patients with HFrEF to improve their quality of life.

## References

[1] Riello III RJ (2021). Heart failure with reduced ejection fraction. Cardiology.

[2] Murphy SP, Ibrahim NE, Januzzi JL Jr (2020). Heart failure with reduced ejection fraction: a review. JAMA.

[3] Speranza Sánchez MO, Quesada Chaves D, Castillo Chaves G (2017). National registry of heart failure in Costa Rica. The RENAIC CR study. Rev Costarric Cardiol.

[4] Ciapponi A, Alcaraz A, Calderón M, Matta MG, Chaparro M, Soto N, Bardach A (2016). Burden of heart failure in Latin America: a systematic review and meta-analysis. Rev Esp Cardiol (Engl Ed).

[5] Armstrong PW, Pieske B, Anstrom KJ (2020). Vericiguat in patients with heart failure and reduced ejection fraction. N Engl J Med.

[6] Kassis-George H, Verlinden NJ, Fu S, Kanwar M (2022). Vericiguat in heart failure with a reduced ejection fraction: patient selection and special considerations. Ther Clin Risk Manag.

[7] Imamura T, Kinugawa K (2022). Initial experience of vericiguat add-on therapy upon fantastic four medical therapy in a patient with systolic heart failure. J Cardiol Cases.

[8] Aimo A, Pateras K, Stamatelopoulos K (2021). Relative efficacy of sacubitril-valsartan, Vericiguat, and SGLT2 inhibitors in heart failure with reduced ejection fraction: a systematic review and network meta-analysis. Cardiovasc Drugs Ther.

[9] Ezekowitz JA, O'Connor CM, Troughton RW (2020). N-terminal pro-B-type natriuretic peptide and clinical outcomes: vericiguat heart failure with reduced ejection fraction study. JACC Heart Fail.

[10] Falco L, Brescia B, Catapano D (2023). Vericiguat: The Fifth Harmony of heart failure with reduced ejection fraction. J Cardiovasc Dev Dis.

[11] Bozkurt B, Hershberger RE, Butler J (2021). 2021 ACC/AHA key data elements and definitions for heart failure: a report of the American College of Cardiology/American Heart Association Task Force on clinical data standards (writing committee to develop clinical data standards for heart failure). Circ Cardiovasc Qual Outcomes.

[12] Heidenreich PA, Bozkurt B, Aguilar D (2022). 2022 AHA/ACC/HFSA guideline for the management of heart failure: a report of the American College of Cardiology/American Heart Association Joint Committee on clinical practice guidelines. Circulation.

[13] Voors AA, Mulder H, Reyes E (2021). Renal function and the effects of vericiguat in patients with worsening heart failure with reduced ejection fraction: insights from the VICTORIA (Vericiguat Global Study in Subjects with HFrEF) trial. Eur J Heart Fail.

